# Prognostic Effect of Microenvironment Phenotype in Triple-Negative Breast Cancer: Biomarker Analysis of a Prospective Trial

**DOI:** 10.3389/fmolb.2021.752154

**Published:** 2021-09-21

**Authors:** Si-Yuan Zhu, Ding Ma, Zhi-Ming Shao, Ke-Da Yu

**Affiliations:** ^1^ Department of Breast Surgery, Fudan University Shanghai Cancer Center, Shanghai, China; ^2^ Shanghai Medical College, Fudan University, Shanghai, China; ^3^ Shanghai Key Laboratory of Breast Cancer, Shanghai, China

**Keywords:** triple-negative breast cancer, microenvironment phenotype, biomarker, trial, immune

## Abstract

**Background:** The microenvironment of triple-negative breast cancer (TNBC) can be divided into three clusters based on bioinformatics-based immunogenomic analysis: the “immune-desert” cluster, the “innate immune-inactivated” cluster, and the “immune-inflamed” cluster. The immune-inflamed cluster is considered as “hot tumor” while the other two are considered as “cold tumor”.

**Methods:** To investigate the prognostic effect of microenvironment phenotypes on TNBC, we compared relapse-free survival (RFS) of different phenotypes in 100 patients with RNA sequencing-based expression data from the PATTERN trial (NCT01216111, published in JAMA Oncol 2020), which indicated a superior efficacy of adjuvant paclitaxel-plus-carboplatin regimen compared to the regimen of cyclophosphamide/epirubicin/fluorouracil followed by docetaxel for TNBC. We also analyzed the efficacy of the two regimens for different immune phenotypes to explore potential treatment strategies.

**Results:** No significant difference in RFS was observed between the “hot tumor” and the “cold tumor” (hazard ratio [HR] = 0.68, 95% confidence interval [CI] 0.28–1.66, P = 0.40). However, the “hot tumor” subtype was associated with significantly longer RFS in node-positive patients (HR = 0.27, 95%CI 0.07–0.97, P = 0.03). Consistently, a similar trend to improved RFS of the “hot tumor” phenotype was detected in patients with stage pT2-3 tumors (HR = 0.29, 95%CI 0.06–1.30, P = 0.08). Furthermore, no significant difference in RFS between the two treatment arms was observed in patients with “hot tumor” (HR = 0.39, 95% CI 0.08–2.01, P = 0.24) or “cold tumor” (HR = 1.05, 95% CI 0.39–2.82, P = 0.92).

**Conclusion:** The microenvironment phenotype in TNBC might have prognostic significance to patients with a high risk of recurrence. The association of the microenvironment phenotypes with the efficacy of adjuvant chemotherapy for TNBC remains to be further studied.

## Introduction

Triple-negative breast cancer (TNBC) accounts for 15–20% of breast cancers that lack estrogen receptor (ER) and progesterone receptor (PR) expression and human epidermal growth factor 2 (HER2) amplification (Perou et al., 2000; Harbeck and Gnant, 2017). Higher risk of relapse and metastasis and lack of therapeutic targets are major problems in TNBC treatment at present (Bianchini et al., 2016; Denkert et al., 2017). Compared with other subtypes of breast cancer, TNBC usually has higher immunogenicity (Lehmann et al., 2011; Burstein et al., 2015). Immune infiltration in the tumor microenvironment (TME) is associated with response to treatment and prognosis of TNBC (Loi et al., 2014; Denkert et al., 2018). Therefore, efforts have been made to explore immunotherapeutic strategies for patients with TNBC. Recent research has shown that the application of immune checkpoint blockade (ICB) may benefit metastatic TNBC (Schmid et al., 2018).

To systemically characterize the impact of the TNBC microenvironment on prognosis and immunotherapy, we have classified the TNBC microenvironment phenotypes into three heterogeneous clusters taking advantage of the expression data of 386 TNBC patients from Fudan University Shanghai Cancer Center (FUSCC): the “immune-desert” cluster with low microenvironment cell infiltration; the “innate immune-inactivated” cluster with resting innate immune cells and nonimmune stromal cells infiltration; and the “immune-inflamed” cluster with abundant adaptive and innate immune cells infiltration (Xiao et al., 2019). The “immune-inflamed” cluster is considered as “hot tumor” while the other two clusters are considered as “cold tumor”.

To further investigate the prognostic effect of the TNBC microenvironment phenotypes and their association with the efficacy of different adjuvant chemotherapy regimens, we conducted a biomarker analysis of the patients with immunogenomic data on microenvironment phenotypes from the PATTERN trial (NCT01216111). The randomized multicenter phase III PATTERN trial compared six cycles of paclitaxel plus carboplatin (PCb) with a standard-dose regimen of three cycles of cyclophosphamide, epirubicin, and fluorouracil followed by three cycles of docetaxel (CEF-T) in the adjuvant setting of operable TNBC, indicating a superior efficacy of the carboplatin-containing regimen compared to the anthracycline/taxane regimen (Yu et al., 2020). A total of 100 patients in the PATTERN cohort with expression data from RNA sequencing or HTA 2.0 microarray have been involved in clustering TNBC microenvironment phenotype mentioned above. Here, we analyzed the clinical characteristics and long-term survival data of these patients to explore clues for potential treatment strategies of adjuvant chemotherapy or immunotherapy for TNBC.

## Materials and Methods

### Study Design

The design and conduct of the PATTERN trial were described elsewhere previously (Yu et al., 2020). In brief, between July 2011 and April 2016, 647 women with operable, primary invasive TNBC after definitive surgery at nine cancer centers and hospitals in China were randomly assigned to two treatment groups: 322 in the CEF-T group and 325 in the PCb group. The primary endpoint was disease-free survival (DFS). Secondary endpoints included overall survival distant DFS, relapse-free survival (RFS), DFS in patients with germline variants in BRCA1/2 or homologous recombination repair-related genes, and toxicity. The independent institutional review board of the participating centers approved the study protocol. We performed the study according to the International Conference on Harmonisation Good Clinical Practice guidelines and ethical principles of the Declaration of Helsinki. All patients provided written informed consent.

### Patient Samples

As mentioned above, a total of 100 patients with RNA sequencing data or HTA 2.0 microarray data in the PATTERN cohort were enrolled in the previous immunogenomic analysis of TNBC microenvironment phenotypes clustering. There were 47 patients in the PCb arm and 53 patients in the CEF-T arm involved, respectively. Detailed inclusion criteria for the analysis were as follows: 1) female patients; 2) unilateral invasive ductal carcinoma; 3) pathologic examination of the ER, PR, and HER2 status performed by the Department of Pathology at FUSCC through immunochemical analysis and *in situ* hybridization (for HER2 status only); 4) patients with no evidence of metastasis at the time of diagnosis; and 5) sufficient frozen tissue for further research. More detailed information regarding the sample processing and sequencing data generation is described previously (Xiao et al., 2019). All data can be viewed in The National Omics Data Encyclopedia (http://www.biosino.org/node) by pasting the accession (OEP000155) into the text search box or through the URL: http://www.biosino.org/node/project/detail/OEP000155. The HTA 2.0 microarray data is also available in GSE76250 and the RNA sequencing data is available in SRP157974.

### Microenvironment Phenotypes Clustering

The detail of microenvironment phenotypes clustering and relevant data processing was described elsewhere (Xiao et al., 2019). In brief, we firstly constructed a compendium of 364 genes to represent 24 microenvironment cell subsets by referring to two gene signatures, CIBERSORT (Newman et al., 2015) and MCP-Counter (Becht et al., 2016). Signatures for types 1, 2, and 17 T helper cells and myeloid-derived suppressor cells were also constructed according to a published article (Angelova et al., 2015). Then we used the “GSVA” function in R to calculate the single sample gene set enrichment analysis (ssGSEA) score to measure the abundance of each cell subset in the samples. Adjusted scores were calculated as the enrichment scores divided by the (1 - tumor purity), which was calculated by the allele-specific copy-number analysis of tumors (Van Loo et al., 2010). Subsequently, k-means clustering was performed to classify the TNBC microenvironment phenotypes into three clusters: the “immune-desert” cluster, the “innate immune-inactivated” cluster, and the “immune-inflamed” cluster. The “immune-desert” cluster and the “innate immune-inactivated” cluster were referred as “cold tumor” while the “immune-inflamed” cluster was referred as “hot tumor”.

### Statistical Analysis

The primary endpoint of this analysis was RFS. The RFS events were defined as the first recurrence of locally, regionally, or distantly invasive disease, a diagnosis of contralateral breast cancer, or death from any cause. The Kaplan-Meier method was used to estimate the distributions of survival outcomes, with the log-rank test evaluating differences of survival outcome. Cox proportional hazards model was used to obtain hazard ratios (HR) and 95% confidence intervals (CI). Differences of continuous and categorical factors were assessed by the Wilcoxon rank-sum test and the χ2 test (or Fisher exact test when necessary). All statistical tests were two-tailed, with the significant level being set at *p* < 0.05. Data were analyzed with STATA version 16.0 and R version 3.4.2.

## Results

### Patient Samples and Clinical Data

**FIGURE 1:**
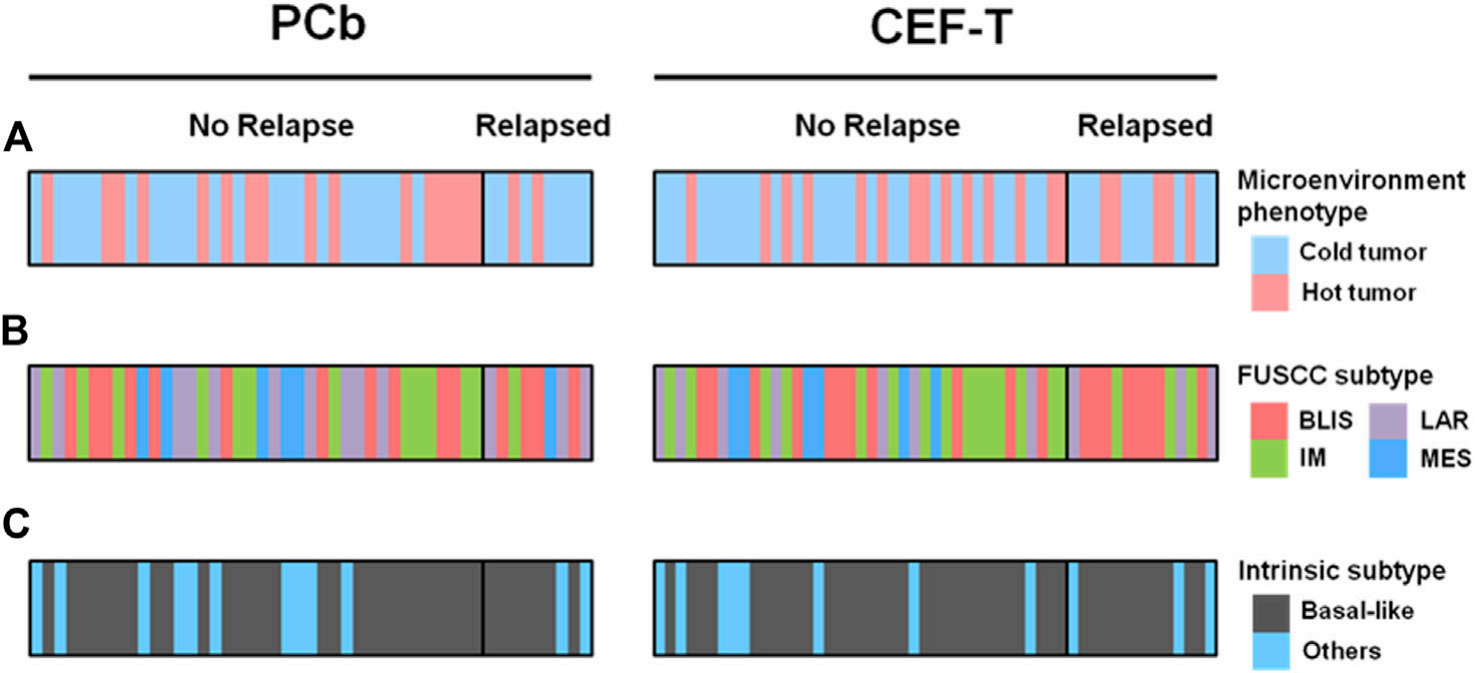
Microenvironment phenotype and other different subtypes by treatment cohorts. **(A)** Microenvironment phenotype, **(B)** FUSCC subtype, and **(C)** intrinsic subtype of the patients enrolled in the analysis. BLIS indicates basal-like and immune-suppressed; CEF-T, fluorouracil, epirubicin, and cyclophosphamide followed by docetaxel; FUSCC, Fudan University Shanghai Cancer Center; IM, immunomodulatory; LAR, luminal androgen receptor; MES, mesenchymal-like; PCb, paclitaxel and carboplatin.

Clinicopathologic characteristics of the 100 patients involved are demonstrated in Table 1. There were 47 patients in the PCb arm and 53 patients in the CEF-T arm, respectively. The median age of these patients was 53 years (interquartile range, 47–59 years) at the time of PATTERN study entry. Among them, 43 patients belonged to the “immune-desert” cluster. Twenty and 37 patients belonged to the “innate immune-inactivated” cluster and the “immune-inflamed” cluster, respectively. Therefore, 63 patients with “cold tumor” and 37 patients with “hot tumor” were included in the analysis. Figure 1 depicted the details of the distribution of the microenvironment phenotype (Figure 1A), the FUSCC subtype (Jiang et al., 2019) (Figure 1B) and the intrinsic subtype (Parker et al., 2009) (Figure 1C) of these enrolled patients.

**TABLE 1:** Clinicopathologic characteristics by microenvironment phenotypes.

Characteristics	Cold tumor (n = 63)		Hot tumor (n = 37)		*p* Value
	No	%	No	%	
Age at diagnosis					
Median (IQR),years	53 (46–61)		53 (49–56)		0.82
Chemotherapy regimen					
PCb	29	46.0	18	48.6	0.80
CEF-T	34	54.0	19	51.4	
Pathologic tumor size					
pT1	26	41.3	19	51.4	0.33
pT2-pT3	37	58.7	18	48.6	
Nodal status					
Negative	41	65.1	18	48.6	0.11
Positive	22	34.9	19	51.4	
Histological grade					
I-II	31	49.2	8	21.6	0.01
III	32	50.8	29	78.4	
Ki67 proliferation index (%)					
≤14	7	11.1	1	2.7	0.13
>14	56	88.9	36	97.3	
Adjuvant radiation					
Yes	16	25.4	15	40.5	0.11
No	47	74.6	22	59.5	

CEF-T, fluorouracil, epirubicin, and cyclophosphamide followed by docetaxel; IQR, interquartile range; PCb, paclitaxel and carboplatin.

Baseline characteristics of the patients of the two types are similar except that patients with “hot tumor” had relatively higher tumor histological grade than the patients with “cold tumor” (P = 0.01). There was no significant difference in chemotherapy regimens for patients of different microenvironment phenotypes as well. Compared with baseline characteristics of the whole PATTERN cohort, more patients enrolled in this analysis were node-positive (Table 2) in that these patients with a relatively greater tumor burden were more likely to provide sufficient frozen tissue and fulfill the criteria for further pathologic examination. 5

**TABLE 2:** Characteristics of the PATTERN cohort and the patients undergoing microenvironment phenotypes clustering.

Characteristics	PATTERN cohort (n = 647)		Microenvironment phenotypes (n = 100)		*p* Value
	No	%	No	%	
Age at diagnosis					
Median (IQR),years	51 (44–57)		53 (47–59)		0.13
Chemotherapy regimen					
PCb	325	50.0	47	47.0	0.55
CEF-T	322	50.0	53	53.0	
Pathologic tumor size					
pT1	351	54.2	45	45.0	0.08
pT2-pT3	296	45.8	55	55.0	
Nodal status					
Negative	481	74.3	59	59.0	<0.01
Positive	166	25.7	41	41.0	
Histological grade					
I-II	177	27.4	39	39.0	0.02
III	470	72.6	61	61.0	
Ki67 proliferation index (%)					
≤14	80	12.4	9	8.0	0.32
>14	567	87.6	92	92.0	
Adjuvant radiation					
Yes	296	45.7	31	31.0	0.01
No	351	54.3	69	69.0	

CEF-T, fluorouracil, epirubicin, and cyclophosphamide followed by docetaxel; IQR, interquartile range; PCb, paclitaxel and carboplatin. Among the patients receiving PCb in this analysis, 20 (42.6%) patients were node-positive and 29 (61.7%) patients were in stage pT2-3. Among the patients receiving CEF-T in this analysis, 21 (39.6%) patients were node-positive and 26 (49.1%) patients were in stage pT2-3. The PCb group had more advanced disease compared with the CEF-T group.

### Prognostic Significance of Microenvironment Phenotypes in TNBC

**FIGURE 2:**
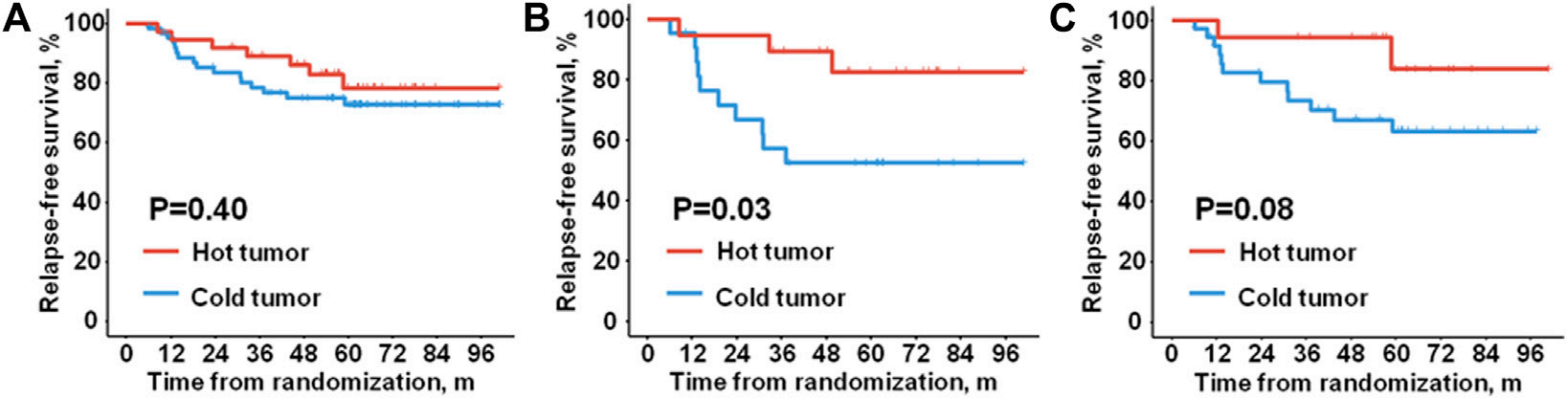
Relapse-free survival of different microenvironment phenotypes. Kaplan-Meier plots show relapse-free survival of **(A)** all the enrolled patients, **(B)** the node-positive patients, and **(C)** the patients with tumors in stage pT2-3.

Considering the important role of TME in tumor progression, we investigated the prognostic significance of different TNBC microenvironment phenotypes taking advantage of the long-term survival data of the 100 patients from the PATTERN cohort. Firstly, we examined the association of microenvironment phenotypes with RFS status. The distribution of different types of the microenvironment in TNBC was similar between patients with different RFS statuses (P = 0.46). No significant difference in RFS was either detected between the patients with “hot tumor” and the patients with “cold tumor” (Figure 2A, HR = 0.68, 95% CI 0.28–1.66, P = 0.40).

However, we found that the TNBC microenvironment phenotypes were significantly associated with RFS status in the node-positive patients (P = 0.04). In contrast, no significant association was found between microenvironment phenotypes and RFS status in the node-negative patients (P = 0.47). Consistently, in the node-positive patients, a significantly better RFS was observed in the patients with “hot tumor” than the patients with “cold tumor” (Figure 2B, HR = 0.27, 95% CI 0.07–0.97, P = 0.03). There was no evidence of different RFS outcomes between the two phenotypes in the patients without lymph node metastasis (HR = 1.57, 95% CI 0.44–5.61, P = 0.48).

Subsequently, we investigated the prognostic relevance of microenvironment phenotypes in patients with different pathological tumor sizes. Given the limited number of cases enrolled in the analysis, a borderline significant association was indicated between the microenvironment phenotype with RFS status in the patients with tumor in stage pT2-3 (P = 0.09). Nevertheless, either of the microenvironment phenotypes was significantly related to RFS status in the patients with tumor in stage pT1 (P = 0.37). Similarly, in the patients with tumor in stage pT2-3, patients of the “hot tumor” phenotype had a borderline significantly longer RFS than the patients of the “cold tumor” phenotype (Figure 2C, HR = 0.29, 95%CI 0.06–1.30, P = 0.08). No significant difference in RFS was observed between the two microenvironment phenotypes in the patients with tumors in stage pT1 (HR = 1.76, 95% CI 0.47–6.57, P = 0.39).

To further validate the reliability of the prognostic effect of the microenvironment phenotypes in TNBC, we conducted a multivariate analysis in patients of different nodal statuses and pathologic tumor sizes (Table 3). Prognostic relevance of the TNBC microenvironment phenotypes in patients with different status of age, histological grades, and adjuvant radiation therapy was also analyzed. The detail of the results was included in the **Supplementary Contents**.

**TABLE 3:** Multivariate analysis of microenvironment phenotypes in TNBC.

	Node-positive			Node-negative			Tumor stage pT2-3			Tumor stage pT1		
Variables	HR	95% CI	*p* Value	HR	95% CI	*p* Value	HR	95% CI	*p* Value	HR	95% CI	*p* Value
Microenvironment phenotype (Hot versus Cold)	0.31	0.08–1.23	0.09	1.78	0.47–6.70	0.40	0.31	0.07–1.51	0.15	1.82	0.40–8.22	0.44
Age (Continuous)	1.01	0.96–1.06	0.75	0.97	0.89–1.05	0.43	0.96	0.91–1.02	0.23	1.07	0.98–1.17	0.12
T stage (pT2-3 versus pT1)	0.92	0.28–3.08	0.89	2.42	0.58–10.08	0.23	N.A.	N.A.	N.A.	N.A.	N.A.	N.A.
Nodal status (Positive versus Negative)	N.A.	N.A.	N.A.	N.A.	N.A.	N.A.	2.50	0.62–10.00	0.20	6.24	1.10–35.34	0.04
Histological grade (Grade I-II versus Grade III)	0.66	0.20–2.18	0.49	0.62	0.15–2.50	0.50	0.65	0.20–2.10	0.47	0.86	0.16–4.70	0.86
Chemotherapy regimen (PCb versus CEF-T)	0.78	0.24–2.50	0.67	0.49	0.11–2.17	0.35	1.02	0.32–3.23	0.97	0.55	0.13–2.44	0.44
Adjuvant radiation (Yes versus No)	0.45	0.14–1.47	0.19	1.30	0.13–13.53	0.83	0.46	0.10–2.03	0.30	0.27	0.04–1.20	0.20

CEF-T, fluorouracil, epirubicin, and cyclophosphamide followed by docetaxel; CI, confidence interval; HR, hazard ratio; N.A., Not appliable; PCb, paclitaxel and carboplatin.

### Microenvironment Phenotypes Relating to the Efficacy of Adjuvant Chemotherapy

**FIGURE 3:**
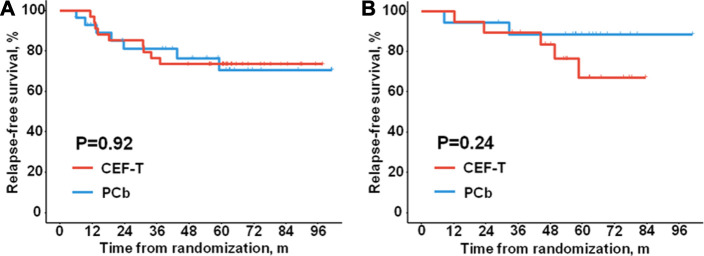
Relapse-free survival of different treatment cohorts. Kaplan-Meier plots show relapse-free survival of **(A)** the patients with “cold tumor”, **(B)** the patients with “hot tumor”. CEF-T indicates fluorouracil, epirubicin, and cyclophosphamide, followed by docetaxel; PCb, paclitaxel and carboplatin.

Considering the different features of genomic alteration of the microenvironment phenotypes in TNBC (Xiao et al., 2019), we further explored the association between the microenvironment phenotypes and the efficacy of adjuvant chemotherapy regimens. In the patients with “cold tumor”, the distribution of RFS was similar in the PCb cohort and the CEF-T cohort (Figure 3A, HR = 1.05, 95% CI 0.39–2.82, P = 0.92). In the patients with “hot tumor”, no significant difference in RFS was detected between the PCb cohort and the CEF-T cohort (Figure 3B, HR = 0.39, 95% CI 0.08–2.01, P = 0.24). Consistently, there was no significant difference in RFS between the patients with “hot tumor” and the patients with “cold tumor” within the PCb arm (HR = 0.39, 95% CI 0.08–1.88, P = 0.22) or the CEF-T arm (HR = 0.99, 95% CI 0.33–2.95, P = 0.98).

Given the prognostic effect of the microenvironment phenotypes in the patients with lymph node metastasis or tumors in stage pT2-3, we examined the association of the microenvironment phenotypes with adjuvant chemotherapy efficacy in these patients who had a relatively higher risk of relapse or metastasis. In the node-positive patients, no significant difference in RFS was observed between the PCb arm and the CEF-T arm no matter in the “hot tumor” subtype (HR = 2.13, 95% CI 0.19–23.63, P = 0.53) or in the “cold tumor” subtype (HR = 0.74, 95% CI 0.21–2.61, P = 0.63). A similar trend of the “cold tumor” phenotype (HR = 1.21 95% CI 0.39–3.75, P = 0.74) was also observed in the patients with tumor in stage pT2-3. RFS events in the “hot tumor” phenotype in patients with tumor in stage pT2-3 were not enough to calculate the HR and 95% CI.

## Discussion

Taking advantage of the expression data of the early-stage TNBC patients from the PATTERN cohort, we investigated the prognostic significance of the microenvironment phenotype in TNBC and its association with the efficacy of adjuvant chemotherapy regimens.

Recent researches have demonstrated that different cell types in the TME are associated with response to treatment and long-term prognosis of TNBC (Denkert et al., 2010; Su et al., 2014; Denkert et al., 2015; Costa et al., 2018). In our study, we found no significant difference in RFS between the patients of the “hot tumor” phenotype and the patients of the “cold tumor” phenotype. However, in the node-positive patients enrolled in the analysis, the “hot tumor” subtype was related to a significantly better RFS compared with the “cold tumor” subtype, while the distribution of survival of the two subtypes was similar in the node-negative patients. This indicates that the “hot tumor” microenvironment phenotype with abundant adaptive and innate immune cells infiltration might be associated with a better outcome for TNBC patients with lymph node metastasis. Consistently, a borderline significantly longer RFS was observed in the “hot tumor” subtype in the patients with tumor in stage pT2-3, suggesting the prognostic effect of the microenvironment phenotypes in the patients with relatively higher tumor burden. By examining the microenvironment phenotypes in TNBC, we can better distinguish the risk of recurrence and metastasis in node-positive patients and high-risk node-negative patients. Yet, no conclusions could be drawn before further validation is conducted in the prospective study.

In addition, as the microenvironment phenotypes in TNBC have a different level of mutation load and homologous recombination deficiency (Xiao et al., 2019), we subsequently explored its association with the efficacy of different adjuvant chemotherapy for TNBC. No significant difference in RFS was observed between the patients treated by PCb and the patients treated by CEF-T in either of the two phenotypes. There was also no significant difference in RFS between the PCb cohort and the CEF-T cohort in the node-positive patients or stage pT2-3 patients of the two phenotypes. It reflects the limited power of the microenvironment phenotypes in TNBC in predicting the efficacy of a carboplatin-containing regimen.

Our research has some limitations. Firstly, the results presented here are limited by their retrospective character despite using a prospective cohort. Secondly, the limited number of cases with microenvironment phenotype data led to the insufficient statistical power of some tests involved. In addition, CEF-T is no longer a standard recommendation in the National Comprehensive Cancer Network guidelines. At present, epirubicin and cyclophosphamide followed by weekly paclitaxel (EC-wP) might be the optimal choice for TNBC (Sparano et al., 2008). Moreover, considering the limited number of cases enrolled in the analysis, larger prospective studies are necessary to determine whether carboplatin can benefit TNBC patients of certain microenvironment phenotypes.

In conclusion, our study reveals that the microenvironment phenotypes in TNBC might predict the prognosis of the node-positive patients and the high-risk node-negative patients. The association of the microenvironment phenotypes with the efficacy of adjuvant chemotherapy for TNBC remains to be further studied.

## Data Availability

All data can be viewed in The National Omics Data Encyclopedia (http://www.biosino.org/node) by pasting the accession (OEP000155) into the text search box or through the URL: http://www.biosino.org/node/project/detail/OEP000155. The HTA 2.0 microarray data is also available in GSE76250 and the RNA sequencing data is available in SRP157974. The data that support the findings of this study are available from the corresponding author upon reasonable request.
