# Supplementary material for: Prognostic Effect of Microenvironment Phenotype in Triple-Negative Breast Cancer: Biomarker Analysis of a Prospective Trial
Source: Front Mol Biosci. 2021 Sep 21;8:752154. doi: 10.3389/fmolb.2021.752154 (PMC8490613; doi:10.3389/fmolb.2021.752154)
Supplement: Supplementary file 1 [file DataSheet1.docx]

**Supplementary content**

**Prognostic significance of the microenvironment phenotypes**

We also analyzed the prognostic significance of the microenvironment phenotypes in triple-negative breast cancer (TNBC) in patients with different status of age, histological grades and adjuvant radiation therapy. In the patients over 55 years old, no significant difference in relapse-free survival (RFS) was observed between the "hot tumor" subtype and the "cold tumor" subtype (hazard ratio [HR]=0.76, 95% confidence interval [CI] 0.20-2.94, P=0.69). Similar trend of RFS was also observed in the patients younger than 55 years old (HR=0.63, 95% CI 0.20-2.06, P=0.45). No significant difference in RFS was either detected between the two phenotypes in the patients with histological grade III (HR=0.99, 95% CI 0.33-2.96, P=0.99) or grade I-II (HR=0.33, 95% CI 0.04-2.60, P=0.27). Similarly, there was either no significant difference in RFS between the "hot tumor" phenotype and the "cold tumor" phenotype in the patients who underwent adjuvant radiation therapy (HR=0.78, 95% CI 0.17-3.47, P=0.74) or those who did not (HR=0.63, 95% CI 0.20-1.94, P=0.41).
